# Stochastic epidemiological model: Simulations of the SARS-CoV-2 spreading in Mexico

**DOI:** 10.1371/journal.pone.0275216

**Published:** 2022-09-29

**Authors:** Pablo Carlos López Vázquez, Gilberto Sánchez González, Jorge Martínez Ortega, Renato Salomón Arroyo Duarte

**Affiliations:** 1 Departamento de Ciencias Naturales y Exactas, Universidad de Guadalajara, Ameca, Jalisco, México; 2 Centro de Investigación Sobre Enfermedades Infecciosas, Instituto Nacional de Salud Pública, Cuernavaca, Morelos, México; 3 Coordinación General de Innovación Gubernamental, Gobierno del Estado de Jalisco, Ciudad Creativa Digital, Guadalajara, Jalisco, México; 4 Coordinación de Análisis Estratégico, Gobierno del Estado de Jalisco, Ciudad Creativa Digital, Guadalajara, Jalisco, México; Affiliated Hospital of Nantong University, CHINA

## Abstract

In this paper we model the spreading of the SARS-CoV-2 in Mexico by introducing a new stochastic approximation constructed from first principles, where the number of new infected individuals caused by a single infectious individual per unit time (a day), is a random variable of a time-dependent Poisson distribution. The model, structured on the basis of a Latent-Infectious-(Recovered or Deceased) (LI(RD)) compartmental approximation together with a modulation of the mean number of new infections (the Poisson parameters), provides a good tool to study theoretical and real scenarios.

## Introduction

Since the late 2019 to date, the rapid worldwide spread of the SARS-CoV-2 has caused around four and a half million of human deaths [1], placing mankind in one of the most challenging episodes in the recent human history. An extraordinary effort has been made to implement mathematical methods to accurately describe the spreading of the epidemic, looking to forecast and to implement non-pharmaceutical responses to reduce the damage in the society [2]. These methods, ranging from standard compartmental models (typically employed to determine the initial epidemiological parameters [3–8]), to hybrid methods that incorporates stochastic meta-population network models with local and global mobility patterns [9–13], attempt to overcome the complex behavior of social interaction characterized by the tendency of the population to cluster [14], following quasi-periodic patterns of mobility in large dense urbanized areas [12, 13, 15]. Furthermore, in addition to the complexity for determining the degree of connectivity among individuals (the contact network), regulatory measures such as home lockdown and social distancing were promoted to reduce the transmission of the infection [1], providing an additional degree of complexity in determining the spreading of the disease.

In this regard, how and when to promote regulatory measures became one of the most difficult decisions to follow, because of their effects in public health, the economy and several other social factors. These decisions had to be supported in predictive models possessing a good equilibrium between registered data (reliable readouts about registered confirmed cases), mobility patterns followed by the population, and computational efficiency of the epidemiological models [8, 11, 13, 16–19].

Moreover, the efficiency of a given model rely on a good accessibility and characterization of the available data [13], which could be more difficult or impossible to be obtained in the case of less developed countries; in this context, stochastic models, which introduce a randomization about certain unknown elements could provide an alternative guidance.

Recently, some stochastic models have been employed to study the Sars-CoV-2 spreading [20–22]; however these models still rely on the law of mass interaction governing the probabilities of infection, an assumption that may not be fulfilled when the dispersion of the disease happens in highly structured social networks following confinement measures; *e.g*., in [20, 21] a compartmental description is used as base model and then additive white Gaussian noise is introduced in the contact parameter *β*; other approximations are carried out by considering a master equation following transition probabilities which also rely on the standard SIR model dynamics and hence a probability of infection proportional to the infected and susceptible populations. In this paper, we introduce a new stochastic model which attempts to overcome the law of mass interaction underlying in the traditional compartmental models. The model has served us to simulate and follow the spreading of the Sars-CoV-2 in Mexico with a good agreement to the real cases; it is structured on the basis of a LI(RD) compartmental model (Latent-Infectious-(Recovered or Deceased)) where the number of infections caused by a single infected individual, per unit time (a day) is randomized, while the daily mean of infected population is modulated through a weight-like time dependent function. The modulation help us to introduce tendencies in the mean of the daily infections caused by several phenomenological or fundamental behavior such as pharmaceutical or non-pharmaceutical interventions and herd immunity as well.

Finally, through this model we analyze the evolution of the disease in some Mexican states (some of them housing the largest metropolitan areas of Mexico), by deriving an empirical approximation of the weight function which is in turn deeply connected to the effective reproduction number R(t).

## Materials and methods

### The model

**Fig 1:**
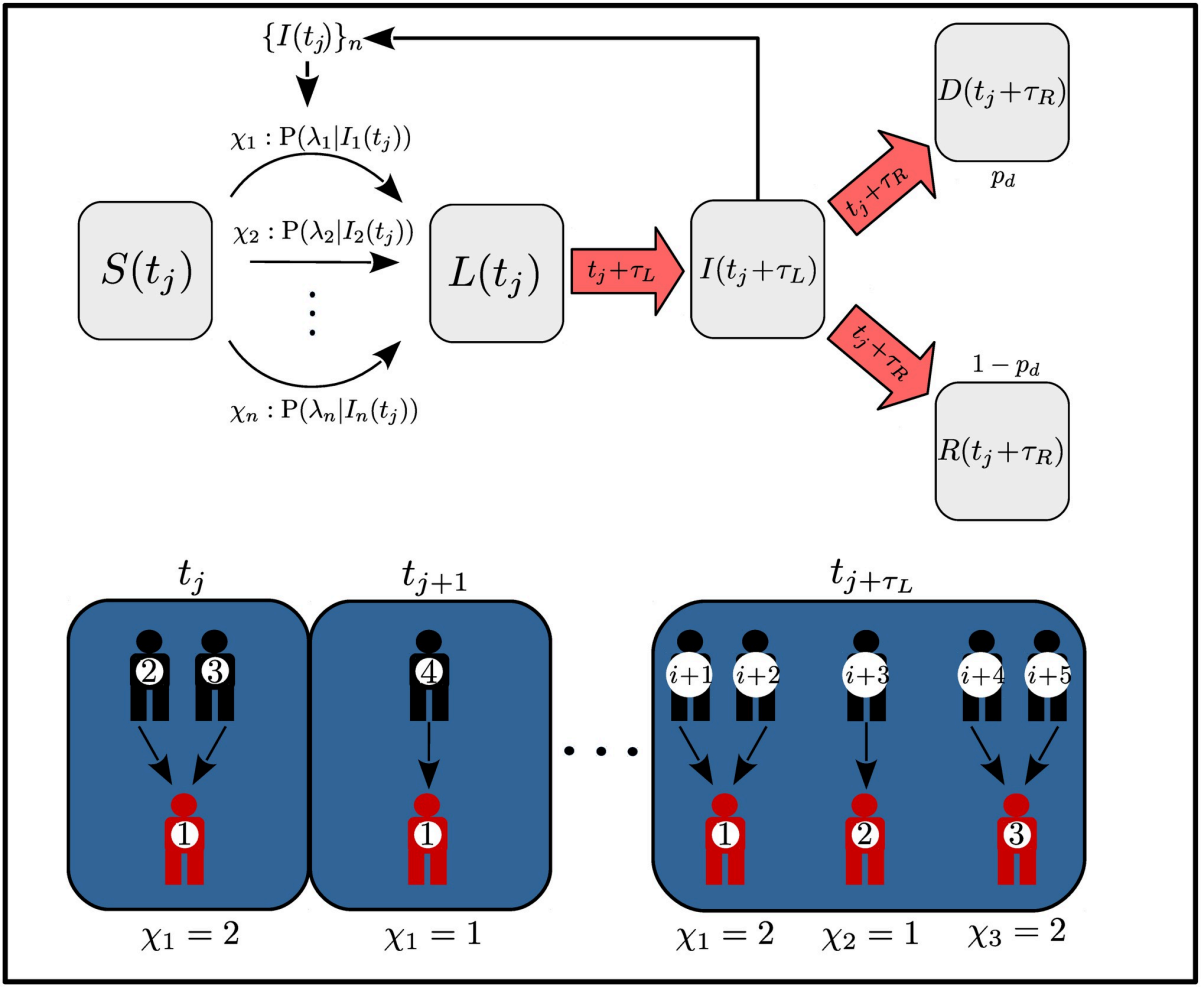
Schematic representation of the model. The figure shows an schematic representation of the stochastic model, where according to the number of infectious population a random number associated to each infectious individual provides the new number of infections. After a latency period, the amount of infected population at time *t*_*j*_ passes into the infectious population generating a new set of random numbers associated to the new infections. Once the recovery period has passed since the beginning of the infections, the infectious population is removed from this condition and moved either to the recovered population with probability 1 − *p*_*d*_, or to the deceased population with probability *p*_*d*_, where *p*_*d*_ represents a probability of decease.

The epidemiological model we propose attempts to describe a scenario about how many people can infect one infectious individual per day when the infectious events are considered homogeneously distributed in time and when the probability of infection is affected by pharmaceutical or non-pharmaceutical interventions; it consists of the randomization of the daily number of infections using a time-dependent Poisson processes to generate the new infections caused by each of the infectious individuals, along a given period of time (the time unit). The core of the model is constructed on the basis of a compartmental description: the susceptible population *S*(*t*) which serves merely to have a finite resource about the number of new infections, the infected-latent population *L*(*t*) which is randomly obtained and the infectious population *I*(*t*) which represents the the part of the population capable of infecting. Once the number of infections per infected individual at a single time step (*i.e*., the daily infected (but not infectious) population per infected individual) has been obtained, they are removed from the susceptible condition an placed into the latent condition *L*(*t*) which characterizes the part of the population that is infected but is not capable of transmitting the virus until an incubation period or latency period *τ*_*L*_, has passed. After the latency period, the infected-latent population becomes contagious, passing into the infectious condition *I*(*t*) and hence becoming able to transmit the disease to the susceptible population by associating to each member of this group a new random process of infection. A schematic representation of the stochastic model is given in Fig 1; in the figure, an infectious population number *n* will generate a set of random numbers following a Poisson distribution associated with infectious individuals and which are summed to the infected population.

The number of the latent and the infectious population at time *t*_*j*+1_ may be written as follows:

$$\begin{array}{ccc} L(t_{j+1}) & = & L\left(t_{j}\right)+\sum_{i=1}^{I(t_{j})}{\left\{\chi \left(t_{j}\right)\right\}}_{i}-\theta \left(t_{j}-t_{L}\right)\;L\left(t_{j}-\tau_{L}\right)\;, \end{array}$$ (1)

$$\begin{array}{ccc} I(t_{j+1}) & = & I\left(t_{j}\right)+\theta \left(t_{j}-t_{L}\right)\;L\left(t_{j}-t_{L}\right)-\theta \left(t_{j}-t_{I}\right)\;I\left(t_{j}-\tau_{I}\right)\;, \end{array}$$ (2)

where *τ*_*L*_ and *τ*_*I*_ represents the period of latency and the period of infectivity, the former defined as the period of time in which an infectious person is capable of transmitting the disease, also, the theta functions *θ*(⋅) are placed in the equations to start counting or removing individuals from the different categories after the latency or infectivity periods have passed. The set of random variables {*χ*(*t*_*j*_)}_*i*_ gives the amount of new infected individuals due to the *i*-th infectious individual at time *t*_*j*_ (which with absolute certainty become infected). The random variables are obtained from a Poisson process, *i.e*.:

$$\begin{array}{c} {\left\{\chi \left(t_{j}\right)\right\}}_{i}\leftarrow \text{Pois}\left(\lambda_{i}\left(t_{j}\right)\right) \end{array}$$ (3)

where the intensities of the Poisson process, (*i.e*. the parameters λ(*t*_*j*_)), describe the mean number of contagious events at the time *t*_*j*_.

In a real scenario, the spreading of a disease depends on the degree of close contact among the individuals and therefore, on the degree of urbanization and mobility of the population [23]; however, we believe that part of these complex aspects could be captured into our model by a proper parametrization of the Poisson processes, *i.e*., the daily mean number of infections per infectious individual λ_*i*_(*t*_*j*_). In this regard, we introduce a time-dependent function which indirectly serves to modify the mean number of the daily infections by associating at each time *t* the following mean of the number of infections produced daily:

$$\begin{array}{c} \lambda_{i}\left(t\right)=\lambda_{i}\;W\left(t\right)\;, \end{array}$$ (4)

where λ_*i*_ represents individual rates of contagious which could be represented as additional random variables assigned to the *i*-th infectious individual following a probability distribution P(*ϱ*_*o*_), *i.e*., {λ}_*i*_ ← P(*ϱ*_*o*_) with parameter *ϱ*_*o*_ representing an initial estimation about the average of the number of infections that a single infectious individual can cause per unit time, *i.e*., the ratio between the basic reproduction number (calculated at the beginning of an epidemiological event [5, 24, 25]) and the infectious period:

$$\begin{array}{c} \varrho_{o}=R_{o}/\tau_{I}\;, \end{array}$$ (5)

additionally, *W*(*t*) in 4 is a time-dependent function (the weight function) which serves to modulate the mean of the number of infections. In other words, the stochasticity of λ_*i*_ attempts to simulate the contact patterns followed by the different individuals of the population given a specific mean of contacts in the whole population while the weight function *W*(*t*) describes modulations about the probability of getting infected, *e.g*., herd immunity, social distancing or confinement. Along this paper we will focus on the case where λ_*i*_ follows a punctual distribution, *i.e*. P(*ϱ*_*o*_) → *ϱ*_*o*_, and we will address the employment of *W*(*t*) in the following subsection.

Finally, and following within the compartment direction, we consider that the infectious population could pass, either to the recovered *R*(*t*) or to the deceased *D*(*t*) condition, depending on the development of the disease in the infected individual (see Fig 1). In the former, we define the recovery period *τ*_*R*_, after which the infected population heals with a given probability of recovering 1 − *p*_*d*_, while the deceased population is the part of the infected population which does not heal according to the decease probability *p*_*d*_. For implementing this procedure we make use of an additional random procedure to randomly select from the infected-latent individuals and according a given fatality rate *l*, the infected population that will pass into the deceased category at the time *t*_*j*_*i.e*., we count every new set of infected-latent individuals appearing at time *t*_*j*+1_ and for each of the new cases, we use a uniform distribution to generate a random number *r* ∈ unif(0, 1) which is compared to *p* = 1 − *l* and if *r* > *p*, we then remove in the future time *t*_*j*+1_ + *τ*_*L*_ + *τ*_*I*_ = *t*_*j*+1_ + *τ*_*R*_ this individual from the infectious condition and place him into the deceased condition *D*(*t*). In this sense, the number of recovered and deceased population at the time *t*_*j*+1_ is given by:

$$\begin{array}{ccc} R(t_{j+1}) & = & R\left(t_{j}\right)+\theta \left(t_{j}-\tau_{I}\right)\;\left(1-p_{d}\right)\;I\left(t_{j}-\tau_{I}\right)\;, \end{array}$$ (6)

$$\begin{array}{ccc} D(t_{j+1}) & = & D\left(t_{j}\right)+\theta \left(t_{j}-\tau_{I}\right)\;p_{d}\;I\left(t_{j}-\tau_{I}\right)\;. \end{array}$$ (7)

Along this paper, we will simulate the evolution of a disease possessing similar epidemiological parameters to those of the COVID-19. We use a basic reproduction number of *R*_*o*_ = 4, an incubation period *τ*_*L*_ = 4 days and an infectivity period of *τ*_*I*_ = *τ*_*R*_ − *τ*_*L*_ = 14 [6, 9, 10, 24–27].

#### Modulation of the Poisson parameters

Without the inclusion of the weight function *W*(*t*), the model we propose represents a probabilistic model with replacements, *i.e*., the probability of infecting a certain amount of susceptible per infected individual would be only determined by a stationary given value, independently of the total population being infected or the contact network. Nevertheless, an intuitive behavior is that as the population of susceptible decreases, then also the chances of having large number of susceptible to fall into close contact with the infectious population; in fact, this is exactly the underlying idea in the emergence of the herd immunity effect. On the other hand, the probability of infection is also continuously changing when contingency measures such as social distancing and home lockdown are implemented in the population. In this regard, an appropriate functionality of the weight function could help us to incorporate such effects.

In the context of an exemplification, we make use of a weight function being the product of a function characterizing the herd immunity effect with an additional function characterizing the variability of the probability of infection along the transients of the epidemiological dynamics due to confinement and other related contingency measures, *i.e*.:

$$\begin{array}{c} W(t)=H(t)\;C(t) \end{array}$$ (8)

where *H*(*t*) represents the herd immunity effect which is estimated to emerge when a large proportion of the population (but not all), has gain certain immunity [28, 29], while *C*(*t*) represents additional changes in the probability of infection along the transients of the dynamics. For the herd immunity effect, we employ a reversed logistic-like function whose argument depends on the fraction of the population that has become infected along the evolution of the epidemic, *i.e*.:

$$\begin{array}{c} H\left(t\right)=\frac{1+\text{exp}(\frac{c_{N}\left(0\right)}{\alpha })}{1+\text{exp}(\frac{c_{N}\left(t\right)}{\alpha })}\left(1-a\right)+a \end{array}$$ (9)

where $c_{N}\left(t_{j}\right)=\sum_{t=t_{o}}^{t_{j}}\sum_{i=1}^{I(t)}\chi_{i}\left(t\right)/N$, is the fraction of the cumulative infected population (Latent and Infectious) at time *t*_*j*_; *α* is a free parameter serving to adjust the stationary value of the infection in the long time limit, and *a* is a lower bound at which the probability of infection is reduced sufficiently to reach the stationarity. In our simulations, we set *a* = 0.1, whereas we have seen that by choosing *α* = 0.22 the herd immunity is achieved when close to the 80% of the population has been infected [29].

**Fig 2:**
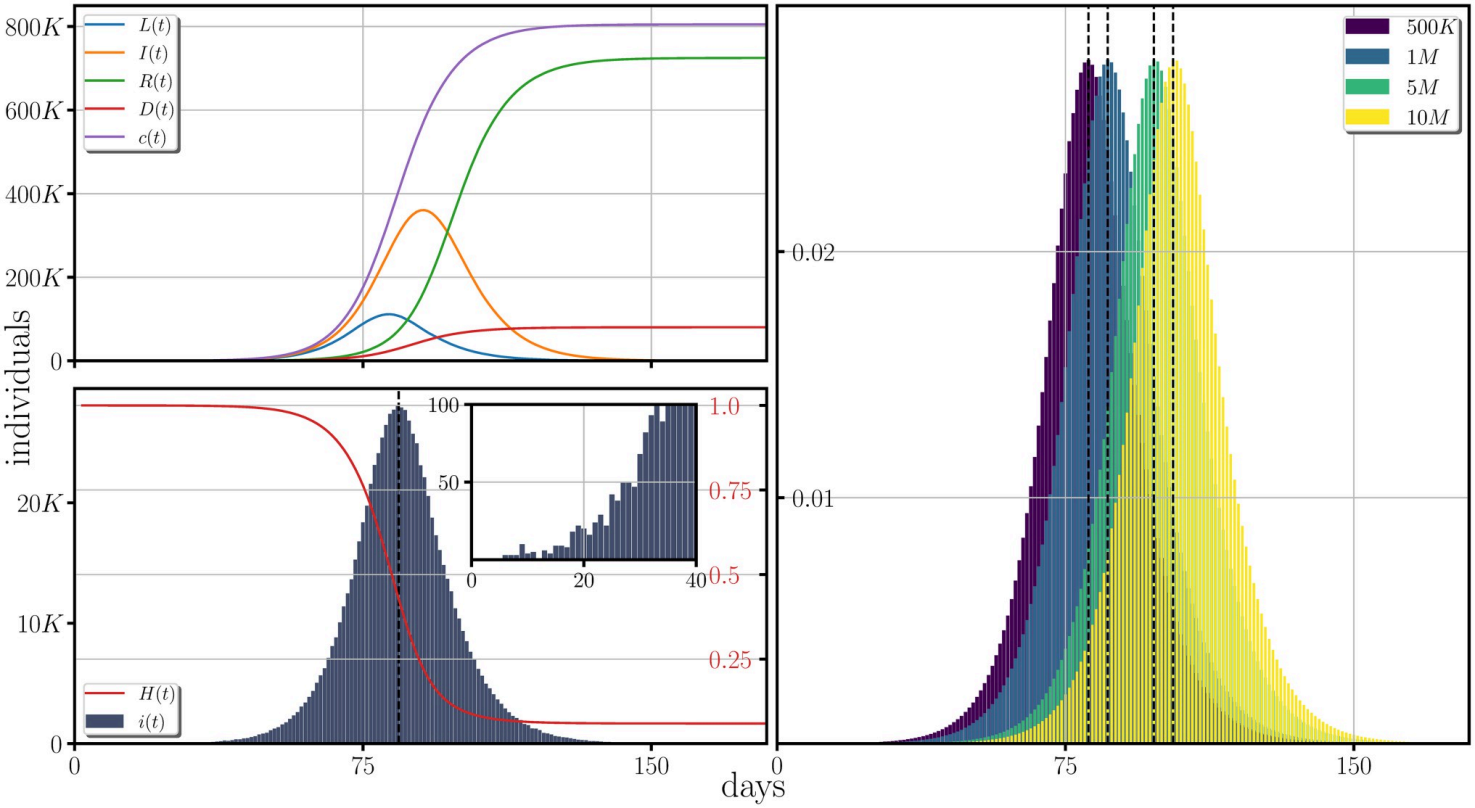
Effects of the weight function representing the herd immunity over the spreading of a disease based on the estimated COVID-19 parameters. At the first column (from left to right), the development of the latent, the infectious, the recovered, the deceased population and the cumulative of the incidence are plotted for a single trajectory. At the left bottom panel is plotted the incidence with the form of the weight function superimposed on the incidence, and representing the herd immunity effect. The y-axis labeled at the right of this panel corresponds to the values taken by *H*(*t*). In the panel at the right, the averages over 5000 trajectories of the normalized incidence for different sizes of the population are shown. The epidemiological parameters employed in the figure corresponds to the estimated values of the COVID-19.

In Fig 2 we present the effect of the weight function to the epidemiological variables when it only characterizes the herd immunity effect (*i.e*.*W*(*t*) = *H*(*t*), *C*(*t*) = 1). In the panels at the left we present single realizations of the latent, the infected, the recovered the deceased, the cumulative of the incidence (top left panel) and the incidence (bottom left panel), together with the form of the weight function generating the herd immunity effect. At the right panel, the normalized incidence is presented for different population sizes and when averaged over 5000 realizations.

The parameters employed in Fig 2 are fixed to the estimated values of the COVID-19 disease described above, although this choice is done only for demonstrative purposes to exemplify the manipulation of the incidence through the weight function and the Fig 2 does not reflects a herd immunity effect emerging in the COVID-19 pandemic.

From Fig 2, one notices that the maximum incidence for a population of one million is obtained from around 3 months after the beginning of the disease spread, reaching at its maximum an amount of roughly 2.5% to 2.7% of the total population. Additionally, if the population is increased in size by one order of magnitude, the maximum is shifted around 20 to 30 days when no contingency measures are implemented in the population.

The effect of non-pharmaceutical strategies to contain the spreading of a disease is another way to modify the probability of infection. In this regard, one could think that some of the most common or intuitive responses of the population under an epidemiological risk: a confinement responding to the daily experience about the development of the disease, *e.g*., a confinement depending upon the number of active cases. In other words, when a certain fraction of the population has become symptomatic-infected (or deceased), it is more likely that some of the susceptible population has knowledge about infected individuals in their social circles or in the neighboring community, reacting with lockdown due to the fear of becoming infected. Another possibility is that contingency measures are placed over the population (typically by health authorities) along different stages of the evolution of the disease, attempting (primarily) to find an equilibrium between the public health resources and different economical activities that require contact among the population. In this case and as we have experienced with the COVID-19 pandemic, all populations have gone through lockdown and relaxation of the confinements during different stages, which in turn, can be imposed at any time of the epidemiological development by the health authorities.

**Fig 3:**
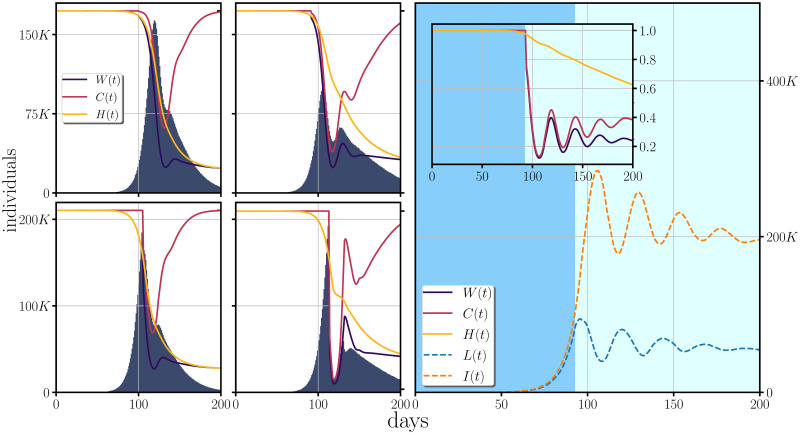
Effect of a confinement based on a Gaussian decay as given in (10) for the COVID-19 parameters. At the four left panels, the incidence is shown with the weight function (containing the effects of herd immunity as presented before, and the effects of the confinement), over-imposed on the incidence. The rows from top to bottom show increasing values of *I*_*o*_: *I*_*o*_ = 5% (top), *I*_*o*_ = 10% (bottom), while the columns from left to right show an increasing decaying rate parameter: *γ* = 5 (left), *γ* = 10 (right). At the right, the figure shows the effect of a strong and early confinement, *γ* = 50 and *I*_*o*_ = 1%, to the latent and the infectious population. In all the cases, the heard immunity is incorporated with the same parameters as used in Fig 2 while we use a total population of 10 million.

We use our stochastic model to explore the behavior of the COVID-19 spreading in two different confinement scenarios, a confinement triggered upon the number of infectious population and an idealized confinement regulated by health authorities at different stages of the dispersion of the disease. In the former, we let *C*(*t*) to be Gaussian-decaying function of the active cases (the infectious population), triggered once certain part of the population has become infectious-symptomatic or deceased, *i.e*.:

$$\begin{array}{c} C\left(t\right)=1+[\text{exp}(-{\left[\gamma \;I\left(t\right)/N\right]}^{2}\;)-1]\theta \left(I\left(t\right)-I_{o}\right) \end{array}$$ (10)

where *I*(*t*) are the infectious cases at time *t*, while *I*_*o*_ represents a threshold about the amount of infectious population at which the confinement function is triggered; *γ* is a decaying-rate parameter describing how strong is the confinement and *N* is the total population. Fig 3 shows the evolution of the disease for a confinement following a Gaussian decay as described in (10). The left four frames represent *I*_*o*_ = 1% and *I*_*o*_ = 10% (from top to bottom) while from the left to the right, *γ* = 5 and *γ* = 10.

In the figure one can see that the outcomes of a confinement relying on the number of the infectious population depend on how strong and rigorous is the confinement and at what stage of the dispersion of the disease is implemented. The different outcomes go from a flattening of the epidemic curve, happening when the confinement does not happens abruptly, to revivals in the incidence which become periodic and more pronounced when the confinement is strong and happens at earlier stages of the epidemic. At the right panel we have plotted the latent and the infectious population under an abrupt confinement (*γ* = 50) at an early stage of infection (*I*_*o*_ = 1%), from which several revivals can be seen. These revivals can be explained by looking at the curve of the latent population: in an abrupt confinement, large part of the population remains on the latent condition and when the number of infectious population is reduced, the latent population will tend to break out the confinement, beginning to produce new contagious events. These results exhibit the need to employ correct times and duration of the confinement measures and that abruptly confinements without proper regulatory measures may trigger revivals.

**Fig 4:**
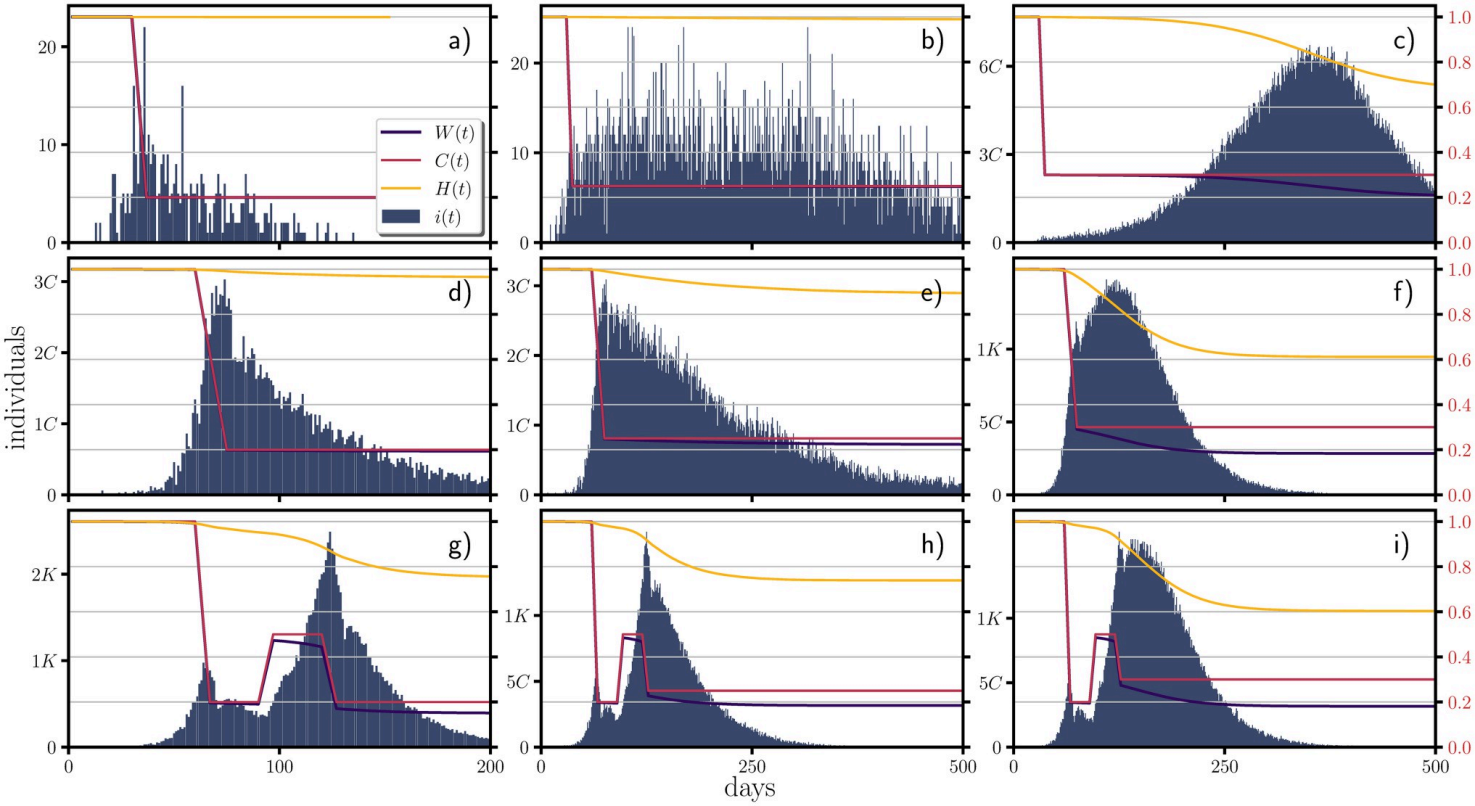
Effects of the piece-wise confinement in the incidence. At the first row (panels a), b) and c)), the confinement begins at the day 30 and the decreasing along 7 days the function *C*(*t*) from its initial value (one) to a 20% of its initial value (panel a)), to a 25% of its initial value (panel b)) and to a 30% from its initial value (panel c)). At the second row (panels d), e) and f)) the confinement begins at the day 60 where the function *C*(*t*) is decreased from its initial value to a 20% of its initial value (panel d)), to a 25% of its initial value (panel e)) and to a 30% from its initial value (panel f)). Finally at the third row (panels g), h) and i)), the function *C*(*t*) is initially decreased to a 20% of its initial value along a period of 7 days, increased back to a value of a 50% of its initial value along a period of 7 days and finally decreased back again at the day 120 along a period of 7 days to a 20% of its initial value (panel g)), to a 25% (panel h)) and to a 30% (panel i)). The y-axis labeled at the right of panels c), f) and i) corresponds to the values taken by *W*(*t*), *H*(*t*) and *C*(*t*). The figure was done using the COVID-19 parameters in a population of one million.

In the context of a confinement based on regulatory measures such as lockdown, social distancing and restrictions on mobility; they could be implemented at any time of the epidemiological development and they will not follow a deterministic behavior (as shown previously). In Fig 4, we explore the generation of the incidence when the probability of infection is manipulated by a piece-wise time-dependent *C*(*t*) function. In this figure, we show single realizations of the behavior of the incidence. In the figure one sees that if confinement is applied at relatively early stages, then a reduction of the *C*(*t*) function below the 25% of its initial value produces a deceleration of the incidence, at 25% the incidence is maintained approximately at a constant rate while anything above the 25% will correspond to increments in the incidence with stronger accelerations for larger values of the *C*(*t*) function.

#### Empirical estimation of *W*(*t*)

Having a self-consistent mechanism that could provide us with an estimation about the evolution of the weight function based on the real available data, would be desirable. We approximate to this problem by using the information accessible through empirical data, such as the empirical incidence *i*_*e*_(*t*) and its cumulative *c*_*e*_(*t*). In our stochastic approximation, the daily synthetic incidence is obtained from a set of random variables following a Poisson distribution; *i.e*. $i_{s}\left(t_{j}\right)=\sum_{j=1}^{I(t_{j})}\chi_{i}\left(t_{j}\right)$, hence the statistical mean of the cumulative of the daily incidence may be written as:

$$\begin{array}{c} {\bar{c}}_{s}\left(t_{j}\right)=\frac{1}{M}\sum_{l=1}^{M}\sum_{k=1}^{j}\sum_{i=1}^{I_{l}\left(t_{k}\right)}\chi_{i}^{\left(l\right)}\left(t_{k}\right) \end{array}$$ (11)

where *M* represents the total number of trajectories to which the statistical mean is performed. If the number of trajectories is large enough, then the statistical mean will approach the expectation value of the random variables, *i.e*., the Poisson parameters associated to the infectious individuals.

Now, if the infection is sustained in the population (*i.e*. the probability of having none new infections is very low), then fluctuations around the mean number of infectious individuals at a given time *t*_*j*_ does not significantly contribute to the averaged incidence over the ensemble; hence by considering the average of the infectious individuals at a given time *t*, *i.e*. $\bar{I}\left(t\right)=1/M\sum_{l}^{M}I_{l}\left(t\right)$, we can approximate the average of the cumulative to:

$$\begin{array}{ccc} {\bar{c}}_{s}\left(t_{j}\right) & = & \sum_{k=1}^{j}\sum_{i=1}^{\bar{I}\left(t_{k}\right)}\lambda_{i}W\left(t_{k}\right). \end{array}$$

In order to gain convergence, we fix as stated before, the Poisson parameters λ_*i*_ to be obtained from a punctual distribution, *i.e*. *P*(*ϱ*_*o*_) → {λ}_*i*_ = *ϱ*_*o*_*δ*_*ii*_ = *R*_*o*_/*t*_*I*_ *δ*_*ii*_, hence we write for the average of the cumulative:

$$\begin{array}{c} {\bar{c}}_{s}\left(t_{j}\right)=\sum_{k=1}^{j}\varrho_{o}\bar{I}\left(t_{k}\right)W\left(t_{k}\right)\;. \end{array}$$ (12)

Our aim is to give an approximate description about the time dependent reproduction number through empirical quantities; in this regard, we do the replacement of the average synthetic cumulative and the averaged infectious population with their correspondent empirical descriptions; ${\bar{c}}_{s}\left(t_{j}\right)\to c_{e}\left(t_{j}\right)$ and $\bar{I}\left(t_{j}\right)\to I_{e}\left(t_{j}\right)$ thus, by expanding the sum to the first steps of propagation, one can recurrently obtain the value of the weight function at the different times, *i.e*.:

$$\begin{array}{ccc} c_{e}\left(t_{1}\right)=\varrho_{o}I_{e}\left(t_{1}\right)W\left(t_{1}\right) & \to & W\left(t_{1}\right)=\frac{c_{e}\left(t_{1}\right)}{\varrho_{o}I_{e}\left(t_{1}\right)} \end{array}$$ (13)

$$\begin{array}{ccc} c_{e}\left(t_{2}\right)=c_{e}\left(t_{1}\right)+\varrho_{o}I_{e}\left(t_{2}\right)W\left(t_{2}\right) & \to & W\left(t_{2}\right)=\frac{c_{e}\left(t_{2}\right)-c_{e}\left(t_{1}\right)}{\varrho_{o}I_{e}\left(t_{2}\right)}\\ & & \vdots \end{array}$$ (14)

$$\begin{array}{ccc} c_{e}\left(t_{j}\right)=c_{e}\left(t_{j-1}\right)+\varrho_{o}I_{e}\left(t_{j}\right)W\left(t_{j}\right) & \to & W\left(t_{j}\right)=\frac{c_{e}\left(t_{j}\right)-c_{e}\left(t_{j-1}\right)}{\varrho_{o}I_{e}\left(t_{j}\right)}\;, \end{array}$$ (15)

therefore, at any given time, one can write for the weight function;

$$\begin{array}{c} W\left(t_{j}\right)=\frac{i_{e}\left(t_{j}\right)}{\varrho_{o}I_{e}\left(t_{j}\right)}=\frac{i_{e}\left(t_{j}\right)}{I_{e}\left(t_{j}\right)}\frac{\tau_{I}}{R_{o}} \end{array}$$ (16)

while the number of infectious individuals at time *t*_*j*_ can be approximated, according the definitions done earlier, as: $I_{e}\left(t_{j}\right)=\sum_{k=0}^{\tau_{I}}i_{e}\left(t_{j}-\tau_{L}-k\right)$.

## Results

### Development of the COVID-19 in some Mexican states

Along the development of the COVID-19 pandemic, we have used the stochastic model to follow the evolution of the spreading of the COVID-19 in certain Mexican states, some being the largest populated states, (Estado de México ∼17 million, Ciudad de México ∼9 million, Jalisco ∼8 million, Nuevo León ∼6 million), housing the largest Mexican metropolitan areas (*i.e*., México City, Guadalajara (Jalisco) and Monterrey (Nuevo León) having populations around 5 to 8 million), and some middle-size populated states (*i.e*., Chiapas ∼5.5 million, Michoacan ∼5 million and Oaxaca ∼4 million) and the state of Nayarit which has a relative small population ∼1 million.

To model the spreading of the COVID-19 in these states, we collect the empirical data of the incidence through the reported cases by the scientific division of the Mexican federal government (CONACyT): https://datos.covid-19.conacyt.mx/ and with that, we construct the empirical weight function as described in (Eq (16)), using the fixed epidemiological parameters of *τ*_*I*_ = 14 days and *R*_*o*_ = 4. Once the empirical weight function is obtained, we generate the synthetic incidence *i*_*s*_ as described in our model (following a Poisson distribution modulated through the weight function). For doing this, we fix an initial time of propagation when the empirical data shows a sustained incidence plus the latency period, and by entering the initial latent individuals (the incidence from the past latency period), we initiate the generation of the incidence by fitting the initial number of infectious individuals such that the averaged trajectories of the synthetic incidence is in good agreement to the empirical data (see the supporting information for reference about the used initial conditions and estimation of the infectious of each case included the empirical incidence used in our simulations).

**Fig 5:**
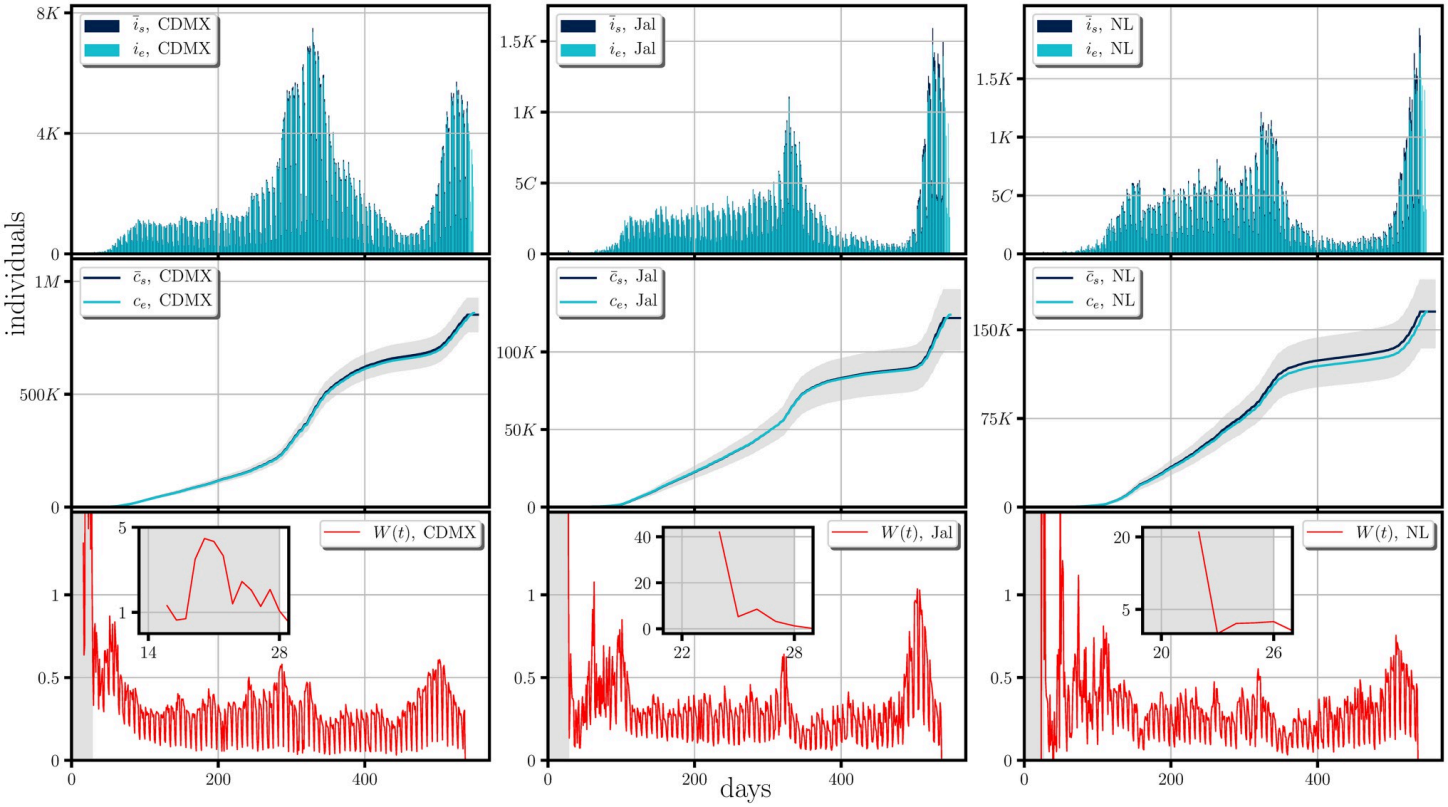
Comparison between the synthetic data generated by the stochastic model and real scenarios happening in some Mexican states and the form of their corresponding weight functions. The figure shows a comparison of the incidence (first row) and its cumulative (second row), between the synthetic data (${\bar{i}}_{s}$ and ${\bar{c}}_{s}$) generated by the stochastic model when averaged over 1000 trajectories to the real scenarios (*i*_*e*_ and *c*_*e*_), happening in Mexico City(CDMX), Jalisco(Jal) and Nuevo León(NL). The shaded region for the cumulative of the incidence represents the 1st and the 3th quartil of the cumulative of the synthetic incidence generated randomly. The synthetic data was generated by employing the empirical estimation of the weight function (last row) obtained form Eq (16). The figure shows a period of roughly a year and a half of the spreading of the SARS-CoV-2 (from February 18th, 2020 to August 20th, 2021).

**Fig 6:**
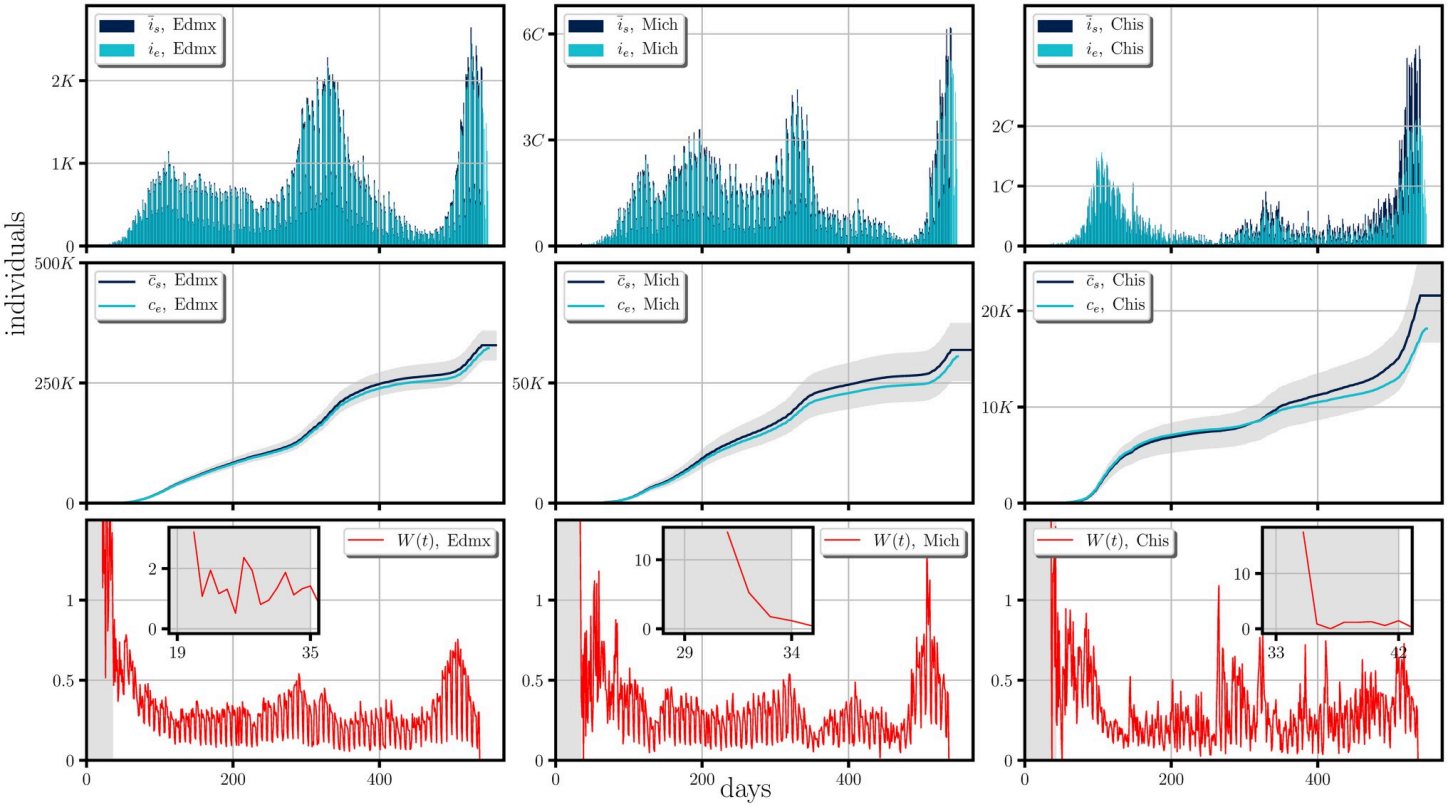
Comparison between the synthetic data generated by the stochastic model and real scenarios happening in some Mexican states and the form of their corresponding weight functions. The figure shows a comparison of the incidence (first row) and its cumulative (second row), between the synthetic data (${\bar{i}}_{s}$ and ${\bar{c}}_{s}$) generated by the stochastic model when averaged over 1000 trajectories to the real scenarios (*i*_*e*_ and *c*_*e*_), happening in Estado de Mexico(Edmx), Michoacan(Mich) and Chiapas(Chis). The shaded region for the cumulative of the incidence represents the 1st and the 3th quartil of the cumulative of the synthetic incidence generated randomly. The synthetic data was generated by employing the empirical estimation of the weight function (last row) obtained form Eq (16). The figure shows a period of roughly a year and a half of the spreading of the SARS-CoV-2 (from February 18th, 2020 to August 20th, 2021).

**Fig 7:**
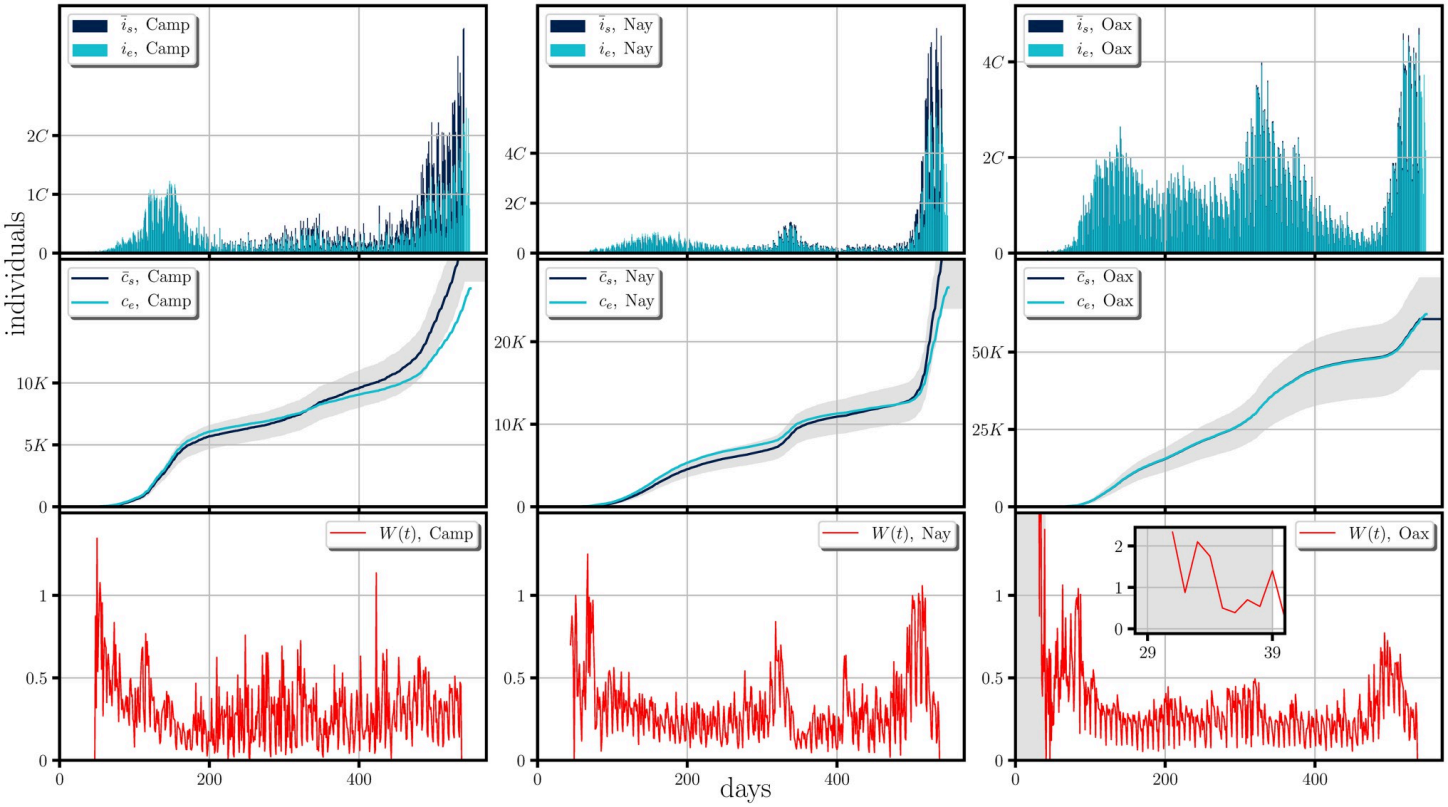
Comparison between the synthetic data generated by the stochastic model and real scenarios happening in some Mexican states and the form of their corresponding weight functions. The figure shows a comparison of the incidence (first row) and its cumulative (second row), between the synthetic data (${\bar{i}}_{s}$ and ${\bar{c}}_{s}$) generated by the stochastic model when averaged over 1000 trajectories to the real scenarios (*i*_*e*_ and *c*_*e*_), happening in Campeche(Camp), Nayarit(Nay) and Oaxaca(Oax). The shaded region for the cumulative of the incidence represents the 1st and the 3th quartil of the cumulative of the synthetic incidence generated randomly. The synthetic data was generated by employing the empirical estimation of the weight function (last row) obtained form Eq (16). The figure shows a period of roughly a year and a half of the spreading of the SARS-CoV-2 (from February 18th, 2020 to August 20th, 2021).

Our simulations are presented from Figs 5 to 7. They show the comparison between the incidence (first rows), the cumulative of the incidence (second rows) and the form of the weight functions for each of the studied cases (third rows), all of them when averaged over 1000 trajectories of the stochastic model. In the general context one can notice same tendencies in the dispersion of the disease and a good agreement to the real cases, with the possible exception of Campeche, (and less noticeable in Chiapas and Michoacan); all the discrepancies starting around the day 350 from the beginning of the dispersion (February 18th, 2020), in which the empirical data lies below our simulations. This discrepancy could be due to i) that the weight function should have taken smaller values than those obtained by the empirical data, which leads us to assume an under-counting on the infectious cases, *i.e*. there were many infectious imported cases (see Eq (16)), or ii) there was an under-counting in the incidence. The former case seems more likely since the discrepancy occurs at a time when the effect of the second wave at a national level has begun to subside hence possible relaxation of mobility measures may have happened.

At the last row of Figs 5–7 we present the form of the weight function for each of the studied cases. The large values of *W*(*t*) shown in the gray shaded areas represent an initial interval of the infection where the occurrence of the incidence, without reported infectious cases require that the majority of the real infectious cases are imported cases while after the shaded area one can expect that the dispersion of the disease relies mostly on the connection network of the local region, the confinement and social distancing regulations.

In this context, one could ask about encoded periodic patterns in the empirical weight function when the local dispersion regime dominates, (*i.e*., the regime at which confinement is well established and imported infectious cases do not contribute largely to the dispersion and estimated in clear areas of the weight function figures (last rows of Figs 5–7)), which could provide us with information about the development of the pandemic *e.g*.the local restrictions, the testing, mobility and the contact network, or even emergent periodic behavior encoded in the probability of infection, during the development of the COVID-19. These periodic patterns can be examined by performing the Fourier transform of the weight function for which, the peaks appearing in the spectrum correspond to frequencies associated to these periodic behaviors.

**Fig 8:**
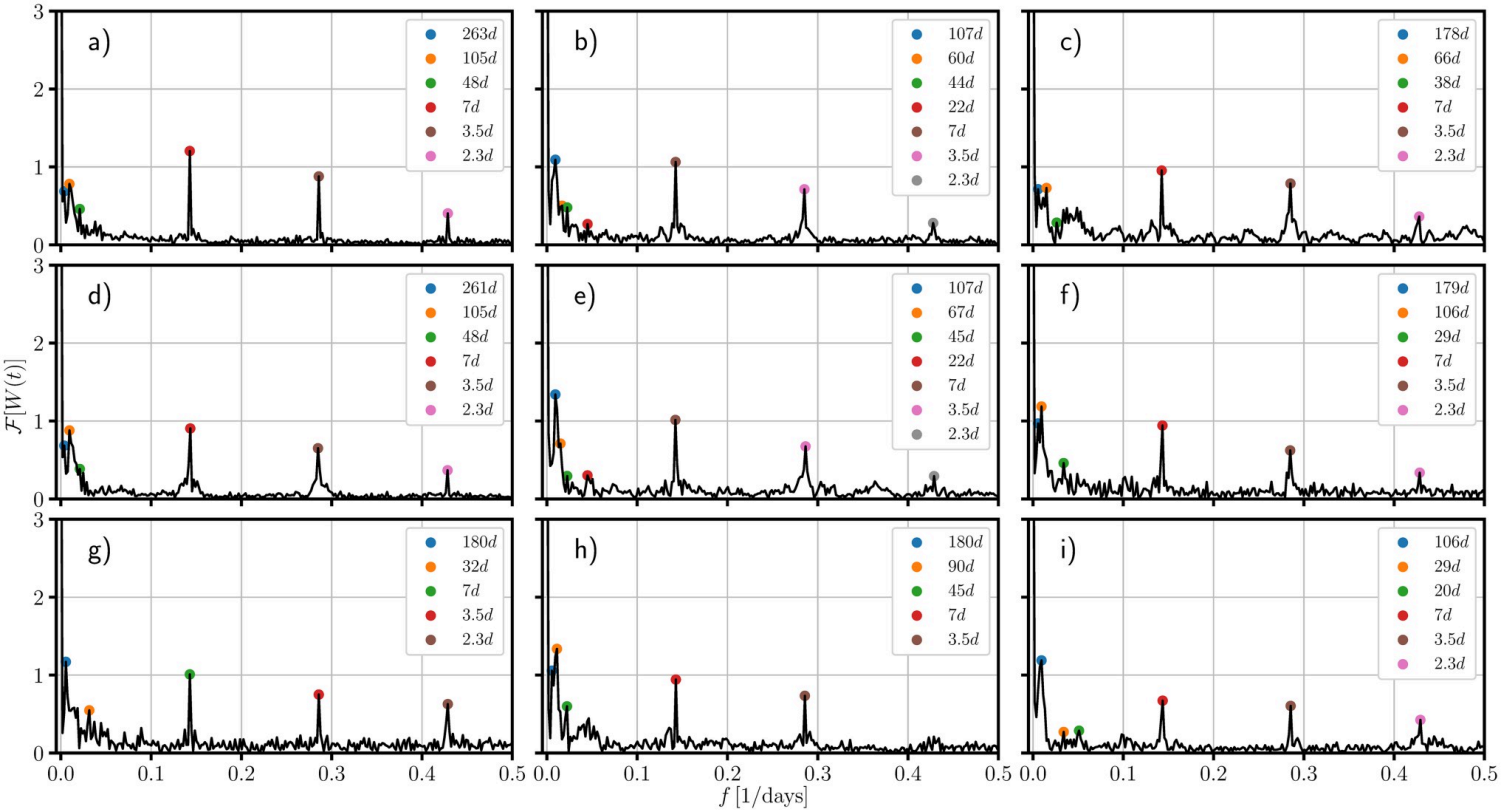
One-sided Fourier transform of the empirical weight function of some mexican federal entities. The figure shows the absolute value of the one-sided Fourier transform of the weight function derived from the empirical weight function of: a) Ciudad de México, b) Jalisco, c) Nuevo León, d) Estado de México, e) Michoacan, f) Chiapas, h) Campeche, i) Nayarit and j) Oaxaca. The colored dots mark the most relevant frequencies associated to periodic patterns in the empirical weight function. The figure shows a period of roughly a year and a half of the spreading of the SARS-CoV-2 (from February 18th, 2020 to August 20th, 2021).

In Fig 8 we present the absolute value of the one-sided Fourier transform of the weight function of each of the studied cases. In the figure we identify different set of frequencies whose corresponding periods (given in days) lie on time scales associated to the weekly agenda, possibly confinements and de-confinements and also to larger patterns such as pandemic waves or even the emergence of seasonality. We identify a first set belonging to periods within a week for which in almost all the cases (with the exception of Nayarit) three peaks are present exactly at the same periods *i.e*. at 7 days, 3.5 days and 2.3 days which may confirm a global (not local) behavior suggesting these are related to the weekly agenda concerning the testing and readouts. A second time scale lies on the range from one two six months which we believe are connected to pandemic waves or periods of confinement, de-confinement and holiday seasons. At this time scale one observes periods that are shared by several states; the periods of roughly six months shared by Nuevo León, Chiapas, Nayarit and Campeche; periods of 3 months shared by Ciudad de México, Jalisco, Estado de México, Michoacan, Chiapas, Nayarit and Oaxaca, and periods of roughly one and a half months which appearing in the states of Ciudad de México, Jalisco, Estado de México, Michoacan and Nayarit. The shared periods could be in some cases explained due to the closeness of certain states (such is the case of Ciudad de Mexico and Estado the Mexico or Jalisco and Michoacan) while other shared peaks between not neighboring states may be telling us something about the connection network of those states. The larger time scale corresponds to a period of roughly 9 months and is represented by the first peak appearing in Ciudad de México and Estado de México. These states share the largest metropolitan area of Mexico (∼ 21 million), hence this pattern suggest a relation to pandemic waves or even the emergence of a seasonal behavior of the COVID-19. This time scale is only present in these states and it may be a consequence of the amount of the population that has become infected, and the large degree of urbanization of this region.

## Discussion

The good agreement between the stochastic model here proposed and the studied cases rely on considering the daily infectious events to follow Poisson distribution, together with the approximations when deriving the empirical weight function; in fact, since the Poisson distribution possess the property that if a set of random variables *χ*_1_, …, *χ*_*n*_, each following independent Poisson distributions with parameters λ_1_, …, λ_*n*_, then the sum of the random variables $\chi =\sum_{i=1}^{n}\chi_{i}$ also follows a Poisson distribution with parameter, $\lambda =\sum_{i=1}^{n}\;\;\lambda_{i}$. In our context, the total number of the daily new infections is a random variable of a Poisson distribution, *i.e*.

$$\begin{array}{c} N\left(t_{j}\right)=\sum_{i=1}^{I(t_{j})}\chi_{i}\leftarrow \text{Pois}(\sum_{i}^{I(t_{j})}\lambda_{i}W\left(t_{j}\right))\;, \end{array}$$ (17)

which, under the assumption that all the individuals have the same probabilities of transmission, (this point refers to the employment of a punctual distribution about the λ_*i*_’s, *i.e*. λ_*i*_ = *ϱ*_*o*_*δ*_*ii*_), then one can state that the probability of having *n* infections at the day *t*_*j*_ should be given by *p*_*n*_(*t*_*j*_) = (*ϱ*_*o*_*I*(*t*_*j*_)*W*(*t*_*j*_))^*n*^ exp(−*ϱ*_*o*_*I*(*t*_*j*_)*W*(*t*_*j*_))/*n*!, or by considering the empirical estimation of the weight function (Eq (16)):

$$\begin{array}{c} p_{n}\left(t_{j}\right)=\frac{i{\left(t_{j}\right)}^{n}}{n!}\;e^{-i(t_{j})}\;. \end{array}$$

*i.e*., the good agreement relies on the fact that we generate the daily incidence from a Poisson distribution whose parameter is the empirical daily incidence. However, in the most general case the assumption of considering same probabilities of transmission for any individual might be difficult to meet and instead one could attempt to connect the weight function to additional empirical quantities. In this regard, one could ask about the relation between *W*(*t*) and an effective reproduction number R(t), the later representing the statistical mean of the infections caused by single individuals once the disease has begun to disperse. To answer this question, lets consider the statistical mean of the number of infected at the time *t*_*j*_ due to the *i*-th infectious individual:

$$\begin{array}{c} N_{i}\left(t_{j}\right)=\sum_{k}{\left[\chi_{i}\right]}_{k}\;p_{k}\left[\lambda_{i}W\left(t_{j}\right)\right]=\lambda_{i}W\left(t_{j}\right) \end{array}$$ (18)

where the [*χ*_*i*_]_*k*_ represents the possible outcomes of the random variable *χ*_*i*_ of the *i*-th infectious at time *t*_*j*_, *i.e*., the possible number of infections that the *i* − *th* infectious could produce with probability *p*_*k*_[λ_*i*_*W*(*t*_*j*_)] of the *k*-th event and λ_*i*_*W*(*t*_*j*_) is the statistical mean of all possible outcomes of the *i*-th contagious individual at time *t*_*j*_. In the case where the Poisson parameters λ_*i*_ are also distributed according to a probability distribution P(*ϱ*_*o*_) (*i.e*., the rate at which each infectious individuals infect is also distributed around a mean *ϱ*_*o*_), then the average of the total number of infected individuals at a fixed time *t*_*j*_ may therefore be given by:

$$\begin{array}{c} R\left(t_{j}\right)/\tau_{I}\equiv \bar{N}\left(t_{j}\right)=\sum_{i=1}^{I(t_{j})}\frac{N_{i}\left(t_{j}\right)}{I(t_{j})}=W\left(t_{j}\right)\;\sum_{i=1}^{I(t_{j})}\frac{\lambda_{i}}{I(t_{j})}. \end{array}$$ (19)

Let us consider now that at certain given time *t*_*j*_ ≥ *t*′, the number of infectious has become large and representative about the dispersion of the disease in the population, *i.e*., the number of infectious can be found homogeneously distributed in the population and the quantity $\sum_{i=1}^{I(t_{j})}\lambda_{i}/I\left(t_{j}\right)$ becomes representative about the mean *ϱ*_*o*_. In other words, for times *t* < *t*′ fluctuations are expected to dominate and as the number of infectious increases, the fluctuations reduce yielding a more localized value of the probability of infection. Therefore, at time *t*_*j*_ > *t*′ one could find a close relation between the weight function defined earlier and the time-dependent effective reproduction number:

$$\begin{array}{c} R\left(t_{j}\right)=R_{o}\;W\left(t_{j}\right)\;. \end{array}$$ (20)

Finally, the stochastic model we are presenting has the advantage that it does not require large computational resources to simulate the dispersion in high populated areas and provide us with a tool to simulate and study idealizations about the changes in the structure of the contact network among the populations. In the context of simulating real scenarios, the success of the model relies on certain information about the evolution incidence, something that cannot be known a priori, although the incorporation of agent-based or complex network models could bring insights about the tendency of the incidence and forecast the spreading of the disease in a given interval of time. Moreover, the shared frequencies shown in Fig 8 suggest a possible synchronization of infectious events between populated areas, revealing deeper complex connections among those regions which could be explored through the implementation of hybrid stochastic-complex network models.

## Conclusion

In this paper we have derived an stochastic compartmental epidemiological model constructed from first principles consisting on a randomization about the number of the new infected population caused daily and following a Poisson process. We have shown that under this assumption, one can reconstruct and simulate the evolution of the development of the COVID-19 pandemic in Mexico, by introducing an additional time-dependent function (the weight function) which is in turn connected to a normalized effective reproduction number. Along this paper, we have focused on the epidemiological parameters corresponding to the COVID-19 disease and through the employment of the weight function, the model is capable of introducing and studying some conceptual behaviors such as herd immunity or certain idealized confinement scenarios. In the former we have employed an inverse-like logistic function of the fraction of the total infected population, which for the COVID-19 epidemiological parameters and without any confinement measures, we have found that the peak of the incidence scales by 20 to 30 days when the total population is increased by one order of magnitude while independently of the population sizes, the maximum incidence reaches from 2.5% to 2.7% of the total population; in the latter, we have explored the reaction of the dispersion of the disease when the population reacts to the infectious population (representing an intuitive reaction of the population under an epidemiological emergence), finding revivals in the incidence (infective waves) if confinement is abrupt and happens at earlier stages in the dispersion of the disease, and a flattening of the epidemic curve on the contrary situation. In this context, we have shown an acceleration of the generation of the incidence when the weight function takes values above 25% of its initial value, a steady behavior in the generation of the incidence for a 25% of its initial value and a deceleration in the incidence when the weight function take values below the 25% of its initial value.

In addition, we have employed our stochastic model together with the definition of the empirical weight function, to simulate the dispersion of the COVID-19 in some Mexican states, some of them housing the major metropolitan areas in Mexico and finding a very good agreement to the real scenarios implying that the infectious events in Mexico could be interpreted as homogeneously distributed events, providing us with an indirect mechanism to estimate dates of super-infectious or anomalous events.

Finally, we have applied the one-sided Fourier transform to the empirical description of the weight function with the intention to look at periodic patterns emerging in the mean of the daily infection which may give us insights about the evolution of the pandemic in Mexico. In this regard, we have found three different set of frequencies corresponding to different time-scales which we identify to a weekly agenda about the capture of the readouts of the testings, confinement and also larger patterns which may be related to pandemic waves or even seasonality.

## Supporting information

S1 File Accession codes. (PDF)

S2 File Repository information. (PDF)

S1 Appendix (PDF)
