# Supplementary material for: Stochastic epidemiological model: Simulations of the SARS-CoV-2 spreading in Mexico
Source: PLoS One. 2022 Sep 29;17(9):e0275216. doi: 10.1371/journal.pone.0275216 (PMC9521938; doi:10.1371/journal.pone.0275216)
Supplement: S1 File — (PDF) [file pone.0275216.s001.pdf]

S1 Accession codes: `https://github.com/RenatoSalArrDu/StochasticSLIRD`
