# Supplementary material for: Stochastic epidemiological model: Simulations of the SARS-CoV-2 spreading in Mexico
Source: PLoS One. 2022 Sep 29;17(9):e0275216. doi: 10.1371/journal.pone.0275216 (PMC9521938; doi:10.1371/journal.pone.0275216)
Supplement: S2 File — (PDF) [file pone.0275216.s002.pdf]

S2 Repository information: We refer the reader to the github directory: `https://github.com/RenatoSalArrDu/StochasticSLIRD` where the supplemental material is located. At this directory, the reader will find:

i) The source codes of the simulations presented in the manuscript

ii) The codes generating the figures of the manuscript

iii) The empirical data used in the simulations presented in the manuscript

iv) A cvs file with the reference about the parameters used in the simulations

i) Simulation codes:

The code used for the simulations was constructed on the basis of fortran 95 language and compiled with the GNU Fortran (GCC) 8.2.0 compiler. The main code and the needed modules are located at the *code* folder having the following characteristics:

- code/main.f95: main code
- code/quartile.f95: module used to compute the quartils of the stochastic trajectories
- code/mconf.f95: module used to implement different forms of the confinement functions (including the empirical description of the weight function)
- code/mcint.f95: module to compute the area under the stochastic trajectories based on montecarlo integration
- code/random.f95: module to generate random numbers from Poisson, uniform, exponential and normal distributions
- code/my_init.f95: module to fix common employed parameters and single and double precision variables
- code/comp.sh: compiling bash script

The code is aided from a bash script (run.sh) which serves to provide code with the parameters of the simulations and to organize and visualize some of the results. This bash script is located at the main github directory and it provides the program the following parameters:

- (if cop = 4), the Mexican federal entity and the final date to simulate (based on the empirical incidence obtained from the federal data bases downloaded from: `https://datos.covid-19.conacyt.mx/`)

- The initial number of infectious, recovered, deceased population, the initial cumulative of the incidence, and the information about previously expected number of latent population.

- The time shift to begin cases from a given date according to the empirical data, the final propagation time and the number of trajectories to simulate

- The epidemiological parameters (in the case of COVID-19 we have fixed these parameters to basic reproduction number: $R_o = 4$, period of recovery: $\tau_R = 18$ days, period of latency: $\tau_L = 4$ and lethality rate $l = 0{,}1$).

- Additional parameters include:

  - Fluctuations around R_o: 1) rop=0, no fluctuations, 2) rop=1, uniform distro fluctuations, 3) rop=2, exp distro fluctuations, 4) rop=2, poiss distro fluctuations
  - Herd immunity simulations (only for wop=0): 1) hop=1, no herd immunity, 2) hop=2, herd immunity based on an inverted logistic function
  - Confinement function: 1) cop=1 No confinement, 2) cop=2 Confinement based on a piece-wise time dependent function, 3) cop=3 Confinement based on a Gaussian decaying weight function: $C(t) = \exp(-(\gamma * i(t))^2)$, 4) cop=4 Confinement based on an empirical description of Rt (simulation of real cases)

For performing simulations the user should download the github directory and input the running location in the run.sh bash script (line number 67), provide empirical data bases to simulate giving the proper location to those files (variable *data* at line 65 of run.sh), and input the corresponding initial parameters. In addition, the empirical data bases used for the simulations presented in the manuscript are located at the code source folder. Finally, the folder "initial_estimation_states"folder located at the source code folder

contains estimated values of the weight function when no empirical estimation but a piece-wise function is used instead (option cop=2)

ii) Figure codes:

The folder named figures located at the main github directory contains the python based codes for generating the figures used in the manuscript; these figures requires of the result of the simulations which are also located at the individual figure folders an are the outcomes of the different performed simulations.

iii) Empirical data:

The empirical data used for the manuscript was downloaded can be accessed in code/Mexico_covid − 19_datas/datas20_08_21. These data were obtained from `https://datos.covid-19.conacyt.mx/` and can be downloaded by any user. The code requires to access this information for generating the empirical weight function and simulations to a further date can be performed by downloading the corresponding data bases, updating the date and the initial parameters in the run.sh script.

iv) Initial parameter reference file:

In the cvs file we have load the empirical incidence obtained from: `https://datos.covid-19.conacyt.mx/` to the date: August the 20th of 2021. In this document, we have included additional columns for each of the analyzed states, giving the cumulative of the incidence and an estimation about the latent and the infectious individuals according to the COVID-19 parameters of ($\tau_L = 4$ and $\tau_I = 14$). In the document we have highlighted what was the initial time of propagation for each state (yellow) together with the latent population (green) and the estimation given to fit the real data about the infectious individuals (red).
