# Supplementary material for: Stochastic epidemiological model: Simulations of the SARS-CoV-2 spreading in Mexico
Source: PLoS One. 2022 Sep 29;17(9):e0275216. doi: 10.1371/journal.pone.0275216 (PMC9521938; doi:10.1371/journal.pone.0275216)
Supplement: S1 Appendix — (PDF) [file pone.0275216.s003.pdf]

S3 Appendix. The standard SIR model limit: In the context of the standard SIR model, the susceptible population S has to be considered corresponding to the part of the population which can be infected. In this regard, its number is constantly reducing due to the contagious events, therefore, according to the random scenario we have proposed, at the time t_{j+1} the number of susceptible can be described as:

$$S(t_{j+1}) = S(t_j) - \sum_{i=1}^{I(t_j)} \chi_i(t_j)\,. \tag{1}$$

with the last term accounting for the part of the susceptible population skipping out from this condition into the latent condition.

From our random model, the standard SIR model can be derived by considering that at any fixed time t_j, any infectious individual infects the same amount of susceptible. This assumptions can be considered valid, (although unrealistic somehow), when the population is homogeneously distributed along the infection area at all time, *i.e.* , there are no clusters of individuals in the population and the reorganization of the susceptible population every time step is homogeneously distributed in space. In this regard, the SIR model limit relies on the assumptions that the distribution of the individuals of the population is independent of the anthropological characteristics of the society.

Following within this idea, we do the replacement of the new daily infected population by each infectious individual by a constant amount $S_o(t_j)$; the total amount of new infected population at the time t_j will yield:

$$\begin{aligned} \sum_{i=1}^{I(t_j)} \chi_i(t_j) &= \sum_{i=1}^{I(t_j)} S_o(t_j) \\ &= S_o(t_j)\, I(t_j)\,. \end{aligned} \tag{2}$$

Moreover, under this assumption, the number of the daily new infected population is scalable in time, hence the population of susceptible can be thought as reservoir of individuals of infinite size such that the epidemic events will not alter the homogeneity of the distribution and one can connect the number of susceptible per unit time $\delta_t = t_j - t_{j-1}$, to the total number of susceptible in the long time limit, *i.e.* $S_o(t_j)/\delta_t = S(t_j)/n\delta_t$. Finally, by redefining the number of the infected population as $L + I \to I$ and by considering the limit of infinitesimal time steps (which will be valid only for

very large population sizes) then, one can write now the set of deterministic equations of the SIR model about the evolution of the disease as:

$$\begin{aligned}
\dot{S}(t) &= -\beta\, S(t)\, I(t), && (3)\\
\dot{I}(t) &= \beta\, S(t)\, I(t) - \kappa_R\, I(t)\,, && (4)\\
\dot{R}(t) &= \kappa_R\, I(t)\,. && (5)
\end{aligned}$$

where contact rate defined as the average number of contacts per individuals per time will be given by $\beta = \text{lím}_{\delta_t \to 0}(n\delta t)^{-1}$ while the recovery rate will fulfill: $\kappa_R = \text{lím}_{\delta_t \to 0}(r\delta_t)^{-1}$, with r being a recovery-time scale factor, *i.e.* $t_R = r\delta_t$. Clearly, for both rates as $\delta_t \to 0$ the quantities $n\delta t$ and $r\delta t$ remain finite.
